# Adapting data management education to support clinical research projects in an academic medical center

**DOI:** 10.5195/jmla.2019.580

**Published:** 2019-01-01

**Authors:** Kevin B. Read

**Affiliations:** Data Services Librarian and Data Discovery Lead, NYU Health Sciences Library, New York University School of Medicine, 577 First Avenue, New York, NY 10016, kevin.read@nyumc.org

## Abstract

**Background:**

Librarians and researchers alike have long identified research data management (RDM) training as a need in biomedical research. Despite the wealth of libraries offering RDM education to their communities, clinical research is an area that has not been targeted. Clinical RDM (CRDM) is seen by its community as an essential part of the research process where established guidelines exist, yet educational initiatives in this area are unknown.

**Case Presentation:**

Leveraging my academic library’s experience supporting CRDM through informationist grants and REDCap training in our medical center, I developed a 1.5 hour CRDM workshop. This workshop was designed to use established CRDM guidelines in clinical research and address common questions asked by our community through the library’s existing data support program. The workshop was offered to the entire medical center 4 times between November 2017 and July 2018. This case study describes the development, implementation, and evaluation of this workshop.

**Conclusions:**

The 4 workshops were well attended and well received by the medical center community, with 99% stating that they would recommend the class to others and 98% stating that they would use what they learned in their work. Attendees also articulated how they would implement the main competencies they learned from the workshop into their work. For the library, the effort to support CRDM has led to the coordination of a larger institutional collaborative training series to educate researchers on best practices with data, as well as the formation of institution-wide policy groups to address researcher challenges with CRDM, data transfer, and data sharing.

## BACKGROUND

For over ten years, data management training has been identified as a need by the biomedical research community and librarians alike. From the perspective of biomedical researchers, the lack of good quality information management for research data [1, 2] and an absence of training for researchers to improve their data management skills are recurring issues cited in the literature and a cause for concern for research overall [1, 3, 4]. Similarly, librarians practicing data management have identified that researchers generally receive no formal training in data management [5] yet have a desire to learn [6] because they lack confidence in their skills.

To address this need, librarians from academic institutions have been working to provide data management education and support to their communities. By developing specific approaches to creating data management education, libraries have found successful avenues in implementing stand-alone courses and one-shot workshops [7], integrating research data management into an existing curriculum [8], and offering domain-specific training [9]. Libraries have offered these training programs by providing general data management training to undergraduate and graduate students [10–12], doctoral scholars [13], and the general research community [14–20], whereas domain-specific data management can be seen most prominently in the life sciences [21], earth and environmental sciences [22, 23], social sciences [24], and the digital humanities [25].

While it is clear that libraries have made inroads into domain-specific areas to provide training in data management, the clinical research community—clinical faculty, project and research coordinators, postdoctoral scholars, medical residents and fellows, data analysts, and medical or doctoral degree (MD/PhD) students—is one that has not received much attention. Clinical research data management (CRDM), an integral part of the clinical research process, differs from the broader concept of research data management because it involves rigorous procedures for the standardized collection and careful management of patient data to protect patient privacy and ensure quality and accuracy in medical care. The clinical research community understands the importance of data standardization [26–29], data quality [30–33], and data collection [28, 34–36] and has established good clinical data management practices (GCDMP) [37] to ensure that CRDM is conducted at the highest level of excellence.

Despite this community-driven goal toward CRDM excellence, there is a dearth of literature about data management training for clinical research, with the only evidence coming from nursing training programs [35, 38], whose research practices are further afield in that they focus on quality improvement rather than clinical investigations. This lack of evidence is surprising considering that the need for CRDM training has been communicated [1, 3, 4, 6].

My library, located in an academic medical center, has supported CRDM through National Library of Medicine informationist projects by collaborating with clinical research teams to improve data management practices [39] and, more recently, by serving as the front line of support for REDCap (an electronic data capture system for storing research data) by offering consultations and comprehensive training [40]. Through REDCap training, I identified a need to expand my knowledge of CRDM to better support the needs of our research community. While REDCap is a tool to help researchers collect data for their studies, the majority of issues that our clinical research community encountered were related to data management. These issues included developing data collection plans, assigning and managing roles and responsibilities throughout the research process, ensuring that the quality of data remains intact throughout the course of the study, and creating data collection instruments. As this recurring thread of issues expanded the learning needs of our community beyond those provided via our REDCap training, I decided to expand my knowledge to address the questions that our researchers asked, to develop a curriculum to support CRDM, and to offer and evaluate CRDM training for our community.

## STUDY PURPOSE

This case study will discuss (a) the development and implementation of a 1.5-hour CRDM workshop for the medical center research community, (b) the results and outcomes from teaching the CRDM workshop, and (c) the next steps for the library in this area.

## CASE PRESENTATION

### Workshop development

#### Gaining skills

Beyond the experience I gained from working closely with researchers on their clinical research projects and through REDCap support, I took two particularly valuable training opportunities that improved my skills in CRDM: the “Data Management for Clinical Research” Coursera course [41] and “Developing Data Management Plans” course [42] offered through the online educational program sponsored by the Society for Clinical Data Management. These two courses provided me with the knowledge that I needed to teach a CRDM workshop but more importantly gave me the confidence to teach it because they provided a depth of knowledge I did not have before. These courses also served to reinforce that the issues and challenges encountered at my own institution were common data management concerns across the broader clinical research community.

#### Identifying core competencies and building workshop content

The primary focus for developing a 1.5-hour CRDM workshop was to use the GCDMP core guidelines [37] as the baseline structure for the workshop. The core guidelines are separated into chapters in the GCDMP, which were used as the foundation for the core competencies of the workshop. Once this baseline structure was established, my goal was to weave in answers to the common questions that our clinical research community has asked through our existing REDCap training. These questions related to how to create codebooks and data dictionaries for research projects, how to structure roles in a research team, how to use best practices for building data collection instruments, how to protect their data according to Health Insurance Portability and Accountability Act (HIPAA) regulations that they should be aware of, how to improve the quality of their data throughout a study, and how to best document procedures throughout a study.

The goal of the workshop was to tie as many examples back to REDCap as possible, because the use of REDCap was written into institutional policy as the recommended tool for research data collection, which made it essential to highlight its data management capabilities. The core competencies combined with the questions mentioned above served as the foundation for developing the learning objectives and interactive learning activities for the workshop (Table 1).

**Table 1:** Clinical research data management workshop core competencies

Core competency	Learning objectives	Interactive learning
Data collection planning	Plan a data collection work flow Document tools and resources used for data collection Connect study protocol to data collection plan	Describe study goal Write down first five steps of the data collection plan Communicate with partner(s)/team to identify gaps
Data collection instrument design	Describe data collection best practices Identify common data collection risks and pitfalls	Review data collection form and identify errors Revise data collection form to collect data according to best practices
Data standards utilization	Define data standards Describe the benefits of using data standards for research Locate data standards for use in research study Navigate the terms of use for specific data standards	Search for relevant data standards in the REDCap Shared Library, National Library of Medicine, and FAIRsharing.org Explain the terms of use for the chosen data standard
Data quality maintenance	Describe the importance of using data quality measures in a clinical research project Implement data quality work flows using REDCap	Develop a data quality plan for an existing or prospective research project Implement the Data Resolution Workflow feature in REDCap
Data storage, transfer, and analysis best practices	Identify institutionally supported data storage and transfer software Identify the components of a statistical analysis plan Describe the documentation needed to perform a successful data transfer	Select the appropriate tool for data storage and transfer based on different scenarios
Role and responsibility management	Describe methods for ensuring that roles and responsibilities are clearly assigned Develop documentation for past, current, and future roles	Assign roles for different project personnel using REDCap Describe methods used to assign roles with partner(s)/team

The core competencies and learning objectives were designed to make the workshop as practical as possible. While the theoretical components of CRDM are important and are emphasized in the workshop, the main focus was to consistently incorporate interactive learning throughout so that attendees could both apply and contextualize what they learned to their own research. Another goal of this workshop was to encourage communication between attendees to highlight common CRDM errors and provide avenues for attendees to learn about successful and unsuccessful approaches from their peers. To this end, after each core competency was taught, the workshop was designed to have attendees discuss their own experiences.

In addition to the core competencies listed in Table 1, the overarching theme and intention applied across the workshop was the importance of maintaining good documentation throughout a clinical research project (e.g., data collection plan, roles and responsibilities documents, statistical analysis plan). By stressing the importance of documentation for each competency, I hoped that attendees would understand the value of and be able to develop their own detailed documentation at each stage of the research process. The time dedicated to developing this workshop—which included reviewing the GCDMP core competencies, outlining commonly asked questions from the research community, establishing learning objectives, building the slide deck, and creating the workshop activities—took between 80 and 100 hours to complete.

### Workshop implementation

The CRDM workshop was offered broadly throughout the medical center three separate times in November 2017, January 2018, and February 2018. These workshops were promoted using our library’s email discussion list of attendees from previous data classes and the Office of Science and Research and Clinical and Translational Science Institute’s announcements emails. Direct outreach was also extended to residency directors and research coordinators, both of whom regularly attend the library’s REDCap training. A fourth workshop was offered in July 2018 as part of the library’s established Data Day to Day series [43], which the library has substantially marketed through posters, write-ups in institutional newsletters, and broadcast emails.

### Workshop evaluation

The CRDM workshop evaluation consisted of both quantitative and qualitative methods using a questionnaire administered at the conclusion of each workshop (supplemental Appendix). This study was deemed exempt by our institutional review board (IRB). Using Likert scales, questions asked attendees to evaluate the difficulty level of the material presented in the workshop, their willingness to recommend the workshop to others, and their intention to use what they had learned in their work. Free-text questions asked attendees to specify how they would use what they learned in their current roles in the institution and what other course topics they would be interested in learning about. For the question that asked attendees to describe how they would use what they learned in their current roles, I hand coded responses in a spreadsheet using the emergent coding technique [44] to identify the competencies that attendees stated as the most applicable to their work.

### Workshop results

**Figure 1:**
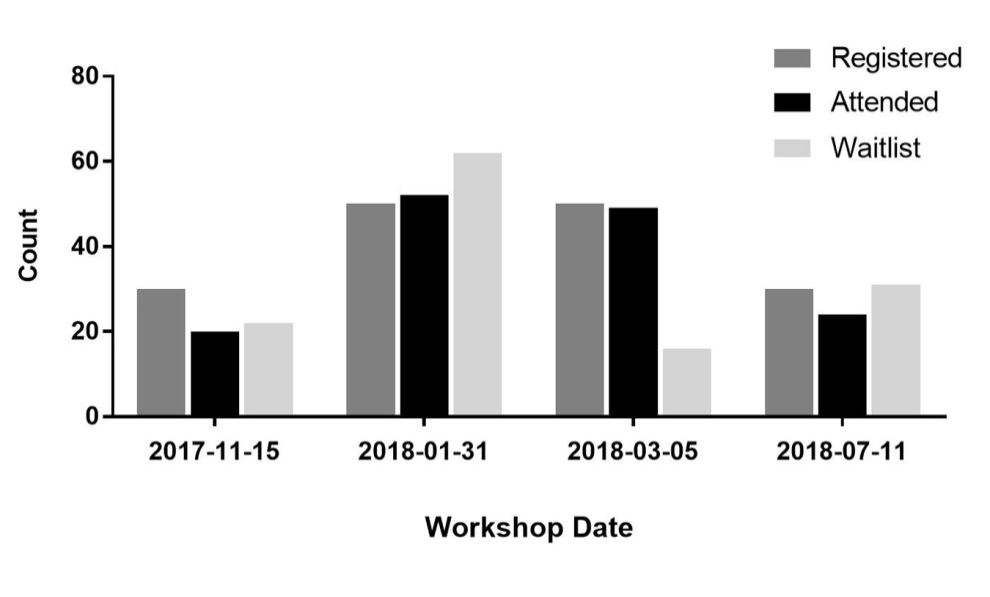
Total attendance, registration, and waitlist numbers for the four clinical research data management (CRDM) workshops

Of the 145 attendees at the 4 workshops, 113 provided fully or partially completed evaluation forms. Overall registration to and attendance at all 4 workshops was very high, with substantial waitlists accumulating for each class offered (Figure 1). In fact, the workshop offered in February 2018 was a direct result of having 60 people on the waitlist from the January session. Waitlists were useful for identifying communities that I had not reached through training to date as well as for understanding the popularity of the topic for the research community. If the waitlist was high in number, it provided another opportunity to offer the workshop or reach out to attendees to see if there was an opportunity to teach a smaller class in their departments.

**Figure 2:**
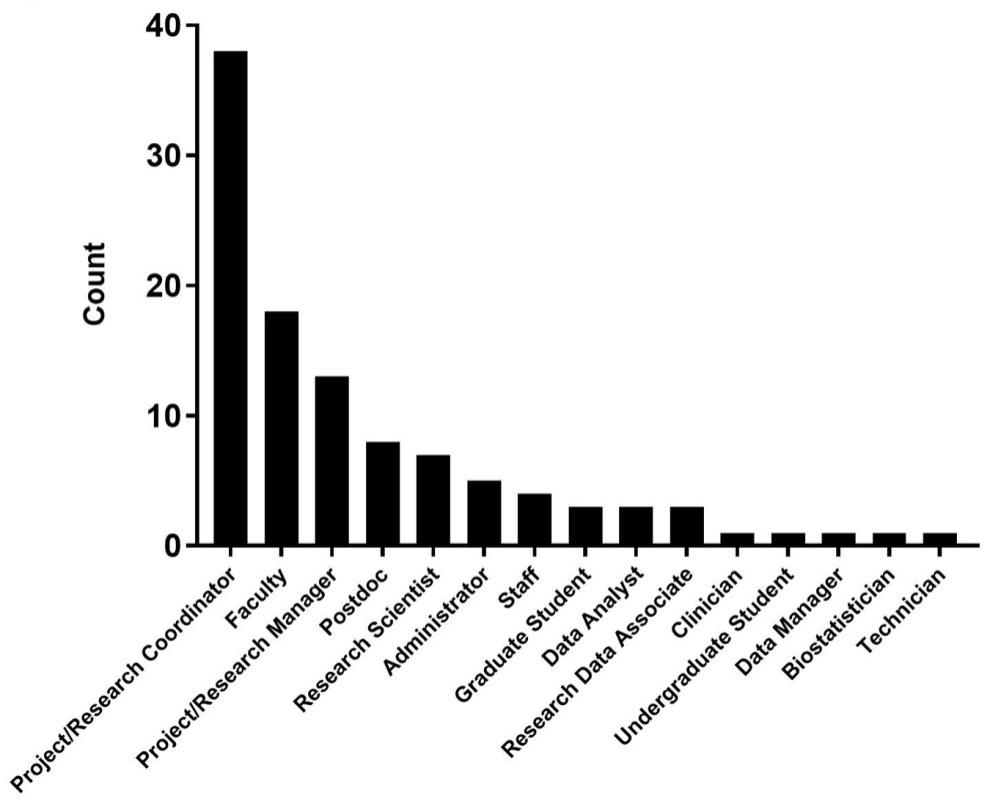
Roles of attendees of the four CRDM workshops

There was a wide range of attendees at these workshops (Figure 2), as there were no restrictions on who could attend. Project/research coordinators (n=38), faculty (n=18), and managers (n=13) were prominent attendees at the workshop, and their comments in the evaluation form reflected its value and the importance of someone from the library teaching this material.

Research coordinators and project managers specifically indicated that the CRDM workshop was helpful in multiple ways for their roles, including how to set up the organization of their data collection procedures, how to establish and clarify roles in a research team, and how to develop documentation for both data collection and the roles and responsibilities of their staff. Research coordinators also indicated that no other stakeholders in the institution taught this kind of material and that this type of training was essential for their work.

Faculty indicated that the workshop was beneficial for developing project management skills, gaining an awareness of the benefits of using REDCap to both collect and manage data, and clarifying the roles and responsibilities of statisticians on their team. They also mentioned the benefits of their study team taking a workshop of this kind at the beginning of a study.

Attendees more generally described the value of the resources presented in the workshop, specifically stating that using REDCap, locating resources for identifying relevant data collection standards, gaining awareness of institutional data storage options, and using the workshop slide deck to guide their CRDM processes were particularly helpful.

**Figure 3:**
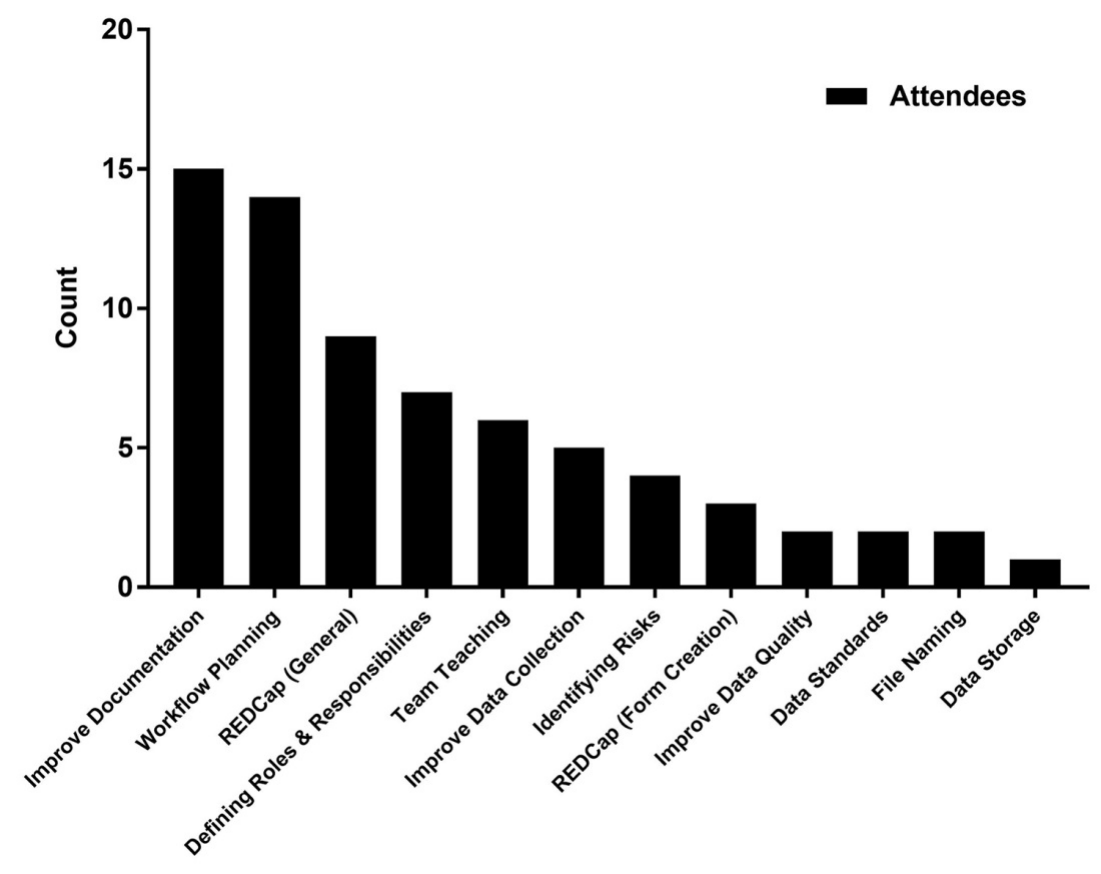
How attendees would use what they learned in their current roles

Overall, the evaluation data indicated positive results, with the majority of those who responded (94%) indicating the level of material was just right and almost all who responded stating they would recommend the class to others (99%) and would use what they learned in their work (98%). Additionally, responses from attendees who indicated how they would use what they learned and apply it to their current role helped provide additional context for the benefits of the CRDM workshop (Figure 3) with improving documentation (37%), planning work flows (34%), using REDCap (22%), and assigning roles and responsibilities (17%) being the most prominent applications of the core competencies learned.

Finally, attendees expressed interest in many additional topics that they would like to see taught in future classes. These topics included statistics, research compliance, the legal implication of data sharing, and IRB best practices for study design. It is important to mention that attendees indicated that they would like to see these additional topics taught in tandem with the CRDM workshop so that they could gain a better understanding of CRDM from the perspective of an established institutional work flow for clinical research projects.

## DISCUSSION

Considering that this was the first time that I had offered CRDM training to our research community, the overall attendance, high waitlist numbers, and percentage of attendees who said the course content was at the appropriate level validated the educational approach that I used. One major concern during the workshop development phase was that the content would be too rudimentary for our research community; however, the evaluations suggested that this was not the case. Furthermore, since one of the central goals of the CRDM workshop was to emphasize the importance of documentation for each core competency, the fact that this was the most commonly cited application of what attendees learned was further validation of the CRDM workshop’s course content.

While my approach was to utilize REDCap as a resource to demonstrate good CRDM practices because it served a direct purpose for our research community, this workshop can be taught without reference to it. The core competencies of this workshop (Table 1) are based on fundamental guidelines of good CRDM practice, and these competencies and skills are applicable to any stakeholder who participates in clinical research, no matter what tool or format they decide to use to collect their data.

The positive reviews of the four broadly offered courses led to seven additional CRDM training sessions that were requested by specific departments and research teams, indicating a strong need from our research community for this material. Evaluation forms were not distributed during these seven sessions due to the consult-like nature of these requests. During these sessions, several research coordinators indicated that the CRDM workshop should be required for all clinical research teams before their studies begin. This call for additional training presents an opportunity for our library to incorporate CRDM education into existing institutional initiatives. Specifically, I identified our institutional education and training management system, residency research blocks, and principal investigator training as logical next steps for integrating CRDM education into institutional research work flows.

The evaluation data initiated the development of partnerships with other institutional stakeholders to better support clinical research training efforts. Our library has begun conversations with stakeholders from research compliance, general counsel, the IRB, the Office of Science and Research, and information technology (IT) to identify ways to better address the needs of clinical researchers. The CRDM workshop highlighted a level of uncertainty on the part of clinical researchers about how best to conduct research in the medical center and whom to contact when faced with certain questions or issues.

Subsequent discussions with the aforementioned stakeholders have emphasized a need to provide more clarity to our community about the research process. To this end, our library is leading the coordination of these groups to offer a comprehensive clinical data education series with representatives from each major department providing their own training to complement the library’s existing REDCap and CRDM workshops. This training series will likely be offered through our library’s existing “Data Day to Day” series so that the research community can take all of the classes within a short time span.

The lack of institutional clarity that attendees and the aforementioned stakeholders identified has also led to policy discussions related to data transfer, sharing, and compliance, as our current institutional procedures are unclear and poorly utilized. Through the development of new standard operating procedures and increased educational initiatives, our library is driving awareness of institutional best practices with the hopes of improving clinical research efficiency. Members from our library now sit on institutional policy working groups that are working to improve institutional data transfer and data sharing work flows.

Just as librarians at the University of Washington carved out a role for themselves in supporting clinical research efforts [45], we seized the opportunity to do the same by offering CRDM education. As the first line of defense for teaching researchers, identifying their data management issues, and hearing their concerns, our library is serving as the conduit for ensuring clinical research is conducted according to GCDM practices at our institution. Establishing partnerships with research compliance, general counsel, the Office of Science and Research, and IT provides us with additional knowledge of their institutional roles and subsequently enables us to send researchers in the right direction to receive the necessary expertise and support. As this service model develops, our library plans to monitor and assess referrals to these other departments to demonstrate the value of increasing compliance in the institution and to integrate CRDM education services into any newly developed policy (which we were successful in doing for the new institutional data storage policy and REDCap). With our library serving as the driving force behind the improvement of CRDM support, the ultimate goal is that these new partnerships will result in our research community being better trained, more compliant, and increasingly aware of established institutional work flows for clinical research

## DATA AVAILABILITY STATEMENT

The workshop evaluation form, resulting data, and slide deck from the “Clinical Research Data Management” workshop are available in Figshare at DOI: http://dx.doi.org/10.6084/m9.figshare.7105817.v1.

## SUPPLEMENTAL FILE

Appendix Evaluation form
