# Supplementary material for: Adapting data management education to support clinical research projects in an academic medical center
Source: J Med Libr Assoc. 2019 Jan 1;107(1):89–97. doi: 10.5195/jmla.2019.580 (PMC6300223; doi:10.5195/jmla.2019.580)
Supplement: Appendix [file jmla-107-89-s001.pdf]

JMLA Journal of the Medical Library Association

SC

Supplemental content to
J Med Libr Assoc. Jan;107(1):dx.doi.org/10.5195/jmla.2019.580
www.jmla.mlanet.org
© Read 2019

Adapting data management education to support clinical research projects in an academic medical center

Kevin B. Read

APPENDIX

Evaluation form

[Class name] [Date] | [Instructor Name]

Affiliation:

- ☐ School of Medicine
- ☐ College of Dentistry
- ☐ Resident
- ☐ College of Nursing
- ☐ Tisch Hospital
- ☐ Other
- ☐ ______________________________

Department/Division: ______________________

Role:

- ☐ Faculty
- ☐ Medical student
- ☐ Resident
- ☐ Project/Research coordinator
- ☐ Graduate student
- ☐ Postdoc
- ☐ Administrator
- ☐ Intern
- ☐ Other: ______________________________

What did you hope to get out of this class?

Will you use what you learned in this class in your work?

- ☐ Definitely will
- ☐ Probably will
- ☐ Probably won't
- ☐ Definitely won't

Was the level of material presented:

- ☐ Too low
- ☐ Just right
- ☐ Too advanced

Would you recommend class to others?

- ☐ Highly recommend
- ☐ Recommend
- ☐ Recommend w/ reservations
- ☐ Not recommend

How will you use what you learned in today's training in your current role?

Interested in adv topics?

- ☐ Yes
- ☐ No

What other topics would you be interested in learning in?

Share any other comments about this class?

